# Infection prevention and control interventions in the first outbreak of methicillin-resistant *Staphylococcus aureus *infections in an equine hospital in Sweden

**DOI:** 10.1186/1751-0147-54-14

**Published:** 2012-03-08

**Authors:** Karin Bergström, Görel Nyman, Stefan Widgren, Christopher Johnston, Ulrika Grönlund-Andersson, Ulrika Ransjö

**Affiliations:** 1 Department of Animal Environment and Health, Faculty of Veterinary Medicine and Animal Husbandry, Swedish University of Agricultural Sciences, SE 750 07 Uppsala, Sweden; 2 Department of Disease Control and Epidemiology, SVA, SE 750 89 Uppsala, Sweden; 3 Equine Clinics, University Animal Hospital, University of Agricultural Sciences, SE 750 07 Uppsala, Sweden; 4 Department of Animal Health and Antimicrobial Strategies, SVA, SE 750 89 Uppsala, Sweden; 5 Department of Clinical Microbiology, Uppsala University Hospital, SE 751 85 Uppsala, Sweden

**Keywords:** Methicillin-resistant *Staphylococcus aureus*, MRSA, Infection control, Basic hygiene, Equine, Outbreak, Environment, Interventions

## Abstract

**Background:**

The first outbreak of methicillin-resistant *Staphylococcus aureus *(MRSA) infection in horses in Sweden occurred in 2008 at the University Animal Hospital and highlighted the need for improved infection prevention and control. The present study describes interventions and infection prevention control in an equine hospital setting July 2008 - April 2010.

**Method:**

This descriptive study of interventions is based on examination of policy documents, medical records, notes from meetings and cost estimates. MRSA cases were identified through clinical sampling and telephone enquiries about horses post-surgery. Prospective sampling in the hospital environment with culture for MRSA and genotyping of isolates by *spa*-typing and pulsed-field gel electrophoresis (PFGE) were performed.

**Results:**

Interventions focused on interruption of indirect contact spread of MRSA between horses via staff and equipment and included: Temporary suspension of elective surgery; and identification and isolation of MRSA-infected horses; collaboration was initiated between authorities in animal and human public health, human medicine infection control and the veterinary hospital; extensive cleaning and disinfection was performed; basic hygiene and cleaning policies, staff training, equipment modification and interior renovation were implemented over seven months.

Ten (11%) of 92 surfaces sampled between July 2008 and April 2010 tested positive for MRSA *spa*-type 011, seven of which were from the first of nine sampling occasions. PFGE typing showed the isolates to be the outbreak strain (9 of 10) or a closely related strain. Two new cases of MRSA infection occurred 14 and 19 months later, but had no proven connections to the outbreak cases.

**Conclusions:**

Collaboration between relevant authorities and the veterinary hospital and formation of an infection control committee with an executive working group were required to move the intervention process forward. Support from hospital management and the dedication of staff were essential for the development and implementation of new, improved routines. Demonstration of the outbreak strain in the environment was useful for interventions such as improvement of cleaning routines and interior design, and increased compliance with basic hygienic precautions. The interventions led to a reduction in MRSA-positive samples and the outbreak was considered curbed as no new cases occurred for over a year.

## Background

A worldwide, steady increase in nosocomial and community-acquired infections due to methicillin-resistant *Staphylococcus aureus *(MRSA) is apparent in humans. Carriage and infections in animals are also reported by many authors [1-7]. The first reported findings of MRSA in equines were in broodmares with endometritis [8,9], but surgical site and traumatic wound infections have been most frequently described [4,10-12]. A special sequence type (ST), 398, has emerged in livestock and horses in European countries [5,7,12-16]. ST398 has also become relatively frequent among MRSA isolates in the human community in some European countries [15-17]. The frequency of MRSA in animals in Sweden is still considered low. MRSA was first detected in Sweden in dogs in 2006 [18] and the first positive horse in Sweden was one nasal carrier found in a MRSA screening study of 300 healthy horses in 2007 [18]. In a recent (2010-2011) screening study of 284 horses, none tested positive for MRSA [19].

In animal hospitals, MRSA is a hazard also for the staff. Colonisation of veterinarians, technicians, students and other people in contact with infected or colonised animals has been reported [20-22]. MRSA is notifiable and subject to mandatory tracing in man, and notifiable in animals in Sweden according to the legislation SJVFS 2007:90. Human and veterinary health authorities collaborate in epidemiological matters. Furthermore, the Swedish Work Environment Authority (SWEA) makes assessments of microbiological hazards by inspections at workplaces and requires any risks to staff to be dealt with [23].

Many outbreaks of MRSA in humans, with their causes, interventions and consequences, have been investigated [1,3,24-29]. The epidemiology of MRSA in equine hospitals has been studied in countries with higher MRSA figures than Sweden [11,12,30]. For the detection of MRSA in the human or animal hospital environment, different sampling and culture methods have been used and evaluated [12,31,32]. Despite this, to the best of our knowledge no standard or recommended method for the environmental screening of MRSA is available. Harmonisation of environmental sampling and culture was attempted in a European Union study of MRSA in dust in pig settings. All participating countries used the same swab technique, culture and typing methods, which were validated [33].

The first outbreak of MRSA in a Swedish equine hospital acted as the trigger to initiate infection prevention and control interventions. Two major interventions have been recommended for the prevention of spread of antimicrobial resistant bacteria in human healthcare, namely infection prevention and control by improved cleaning, better hand hygiene and clothing routines, and restriction of antimicrobial use [34-36]. In Sweden, discussions and awareness on restricting the use of antibiotics in animals were fairly well established before the referred outbreak [18,19], while basic hygiene in equine settings has been less thoroughly discussed. In this study we therefore focused on infection prevention and control.

The aim of the present study was to describe and discuss infection prevention and control measures undertaken to curb an outbreak of nosocomial MRSA infection and achieve long-term improvement of infection prevention and control in a Swedish equine hospital.

## Methods

### Study design

This was a descriptive study of interventions based on retrospective examination of policy documents, medical records, notes from meetings and cost estimates generated by the first MRSA infections in horses in Sweden. Prospective environmental sampling for MRSA with genotyping of isolates by *spa*-typing and pulsed-field gel electrophoresis (PFGE) was performed from July 2008 to April 2010 to investigate potential spread of an outbreak strain within the hospital.

The study has approval, C 120/7, by the Ethical Committee on Animal Experiments, Uppsala, Sweden. The study met the existing Swedish laws and legislations; MRSA is notifiable in animals according to SJVFS 2007:90 and in humans notifiable and subjected to mandatory tracing according to the law SFS 2004:168 and regulation 2004:255.

### The outbreak

**Figure 1:**
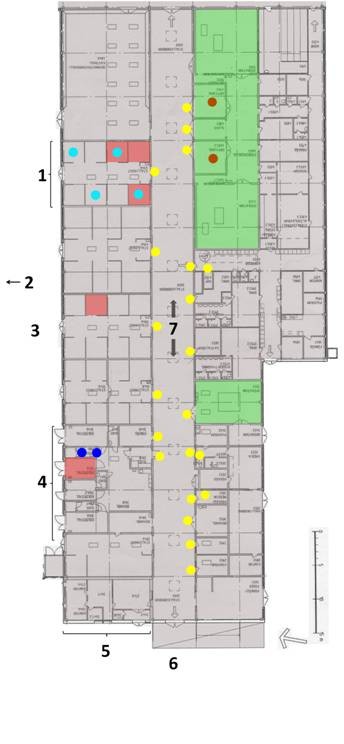
**Surfaces testing positive for MRSA in ward A of the equine hospital**. Samples from July 2008. Dots denote positive environmental samples: Yellow - door knobs, Blue - ISO, Turquoise - cribs and salt stones, Brownish-red - sling/traverse (see also Table 2). Some dots represent pooled samples e.g. in the lameness run-up, one cloth was used in 21 door knobs making one pooled sample and in Stall A several salt stones + cribs were sampled with one cloth. Green area is the surgery unit. The smaller green area is 'room 3' for standing surgery procedures. The pinkish-brown squares are stalls housing MRSA-positive horses during the outbreak. 1 - Stable A, 2 - arrow towards ward B, 3 - stable yard, 4 - isolation unit in ward A, 5 - front desk, 6 - main entrance, 7 - lameness run-up corridor.

The outbreak is described in detail by Bergstrom et al. [37] and is only briefly summarised below. Six horses suffered from surgical site infections (SSI) due to MRSA ST398 *spa*-type t011 during a two-month period from the end of May to July 2008 in an equine hospital in Sweden. Genotyping by PFGE confirmed the outbreak by showing that all isolates were of the same origin. Case 1 underwent surgery on 22 May (Table 1) and Cases 2 and 3 were operated within the following 10 days to early June. Cases 1-3 had indirect contact through operating theatres or surgical equipment [37]. Cases 4, 5 and 6 underwent surgery within a six-day period in the first week of July. Case 6 was an emergency and the only horse that underwent surgery after the second case was detected and an outbreak was suspected. Post-operative horses, except Case 6, were housed in Stable A after surgery (Figure 1). Case 1 was euthanized in the hospital on June 2 and Cases 2, 3, 4 and 5 were discharged. Case 6 remained in the isolation unit until mid-December. Case finding through tracing of asymptomatic contacts with nasal screening in the home stables of Cases 1, 2 and 3 was decided upon by the Swedish Board of Agriculture (SJV) in July.

**Table 1:** Course of the MRSA outbreak and interventions in an equine hospital, 2008-2010

Time	Course and Intervention
**2008**	

**Jan**	***Jan 1, Methicilin-resistant coagulase-positive staphylococci notifiable in animals in Sweden*.**

**June**	**12 June**, ***MRSA Case 1 ***diagnosed. Surgery on May 22.

**July, main outbreak month**	**4 July, *Case 2 ***diagnosed (surgery on 2 Jun). Outbreak suspected. Five of six horses infected had undergone surgery at this time***. Elective surgery suspended ***and Stable A closed. ***First telephone meeting ***with human and veterinary authorities.
	**16 July, *Meeting 1 ***- at the UDS with community vet, public health nurse + SVA.
	***Tracing ***- 45 horses had surgery during outbreak, 37 answers, four suspects, one MRSA-positive.
	**18**-**30 July*, Case 3, 4 and 5 ***diagnosed, sampled at home as clinical infection. Case 5 traced by phone.
	**22 July*, first environmental sampling***, 7 of 18 positive (7/18) (Table 2). Door knobs disinfected.
	***Consultation visit*, **human infection control (IC). IC committee + executive working group formed.
	***Disinfectant dispensers ***installed at all sinks and doors.
	***Replacement ***of water feeders with buckets started.

**Aug**	**16 Aug**, ***Case 6 ***diagnosed, emergency surgery on 7 July, stayed isolated in UDS until mid-Dec.
	***Meeting 2 ***- IC consultation, county vet, heads of UDS, further discussions + planning.
	***Meeting 3 ***- with staff concerning sampling of personnel and education of basic hygiene.
	***Cleaning of surgery unit ***- theatres cleaned by professional hospital cleaners.
	***Cleaning routines ***- critical points tenside + isopropylalcohol disinfection, cleaning detergent only.
	***Implementation ***- e.g. surgery routine, no brush of skin, soap + alcohol-based hand disinfect.
	***Equipment test ***- endoscope (culture working canal), arthroscope, disinfector, autoclave.
	***Elective surgery restarted***.

**Sep - Nov**	***Basic hygiene ***policy + personal appearance, for staff ***to read and sign***.
	***Lecture ***- to staff about MRSA, hygiene, infection control and the implementation process.
	***Voluntary MRSA sampling ***of staff, 12/30 sampled, all neg.
	***Cleaning ***- elimination of loose equipment and cleaning of stables.
	***Replacing ***- most water feeders + cribs replaced by buckets. Salt stones + holders abolished.
	***Second environmental sampling ***- 1/14, on pooled sample from 21 door knobs pos (Table 2).

**Dec**	***Third environmental sampling - retest ***of door knobs divided into seven samples, 0/7 (Table 2).
	***Written routines ***- surgery unit including hygiene, cleaning, instrument washing + disinfection and preparing horses for surgery.
	***Written report ***from IC consultant, what had been achieved and what remained to be done.

**2009**	

**Jan - Feb**	***Renovation ***- recovery rooms new floor/walls, flusher disinfector installed.
	***Written routines ***for cleaning and disinfection of stables, day patient area + ISO.
	***Written routines ***disinfection of flexible endoscopes.

**Mar - July**	***Meetings ***- staff, lectures as in Sept 2008 + implementation.
	***Fourth environmental sampling ***- 1/17, hand touch surface surgery unit pos (Table 2).
	***Written routines ***for ISO area revised.

**Sep**	***MRSA case ***- pressure sore, foal kept in ISO.
	***Fifth environmental sampling ***- 0/11 (Table 2).

**Oct**	***Feedback ***- staff lecture, discussion hygiene policy.
	***Sixth environmental sampling ***- 0/5 (Table 2).

**2010**	

**Jan**	***Feedback ***- staff, feedback on pilot study and infection control/basic hygiene discussions

**Mar**	***MRSA case ***- SSI, horse kept in ISO.
	***Seventh and eighth environmental sampling ***- 0/15 and 0/3 respectively (Table 2).

**Apr**	***Ninth environmental sampling ***- ISO, 1/3, stall pos (Table 2).
	***Continuing work; Revaluation, revision of routines, education, implementation etc*.**

After the outbreak in 2008, two more MRSA-infected horses were detected in the hospital during the study period (Table 1). A foal admitted to the hospital for neonatal care in the last week of June 2009 revisited in August for minor surgery without complications, but on a third visit in September 2009 MRSA was detected in a pressure wound caused by bandages. In February 2010 a horse underwent surgery and was readmitted with an MRSA infection.

### Setting at the time of the outbreak

The outbreak occurred in the Equine Clinics (UDS) at the University Animal Hospital, Swedish University of Agricultural Sciences. A large animal ambulatory service and small animal clinics were housed in the same building complex as the UDS. The section for diagnostic imaging, which served all clinics, was adjacent to the UDS. The UDS offered advanced medical, surgical, orthopaedic and intensive care and specialist clinics. A maximum of 42 horses could be accommodated at the hospital at one time.

Regular opening hours were 8 a.m. to 4 p.m. The emergency unit accepted calls until 12 p.m. and 24-h critical patient care was provided. Five to six veterinarians and 12 to 15 technicians worked in the hospital during the regular opening hours. One or two veterinarians and technicians worked the evening shift. Up to eight veterinary students at a time and teachers were involved in clinical work during teaching periods, approximately from mid-August to mid-December and mid-January to mid-June. Most of the staff had long working experience but less formal training in infection control.

The building that houses the UDS was built in the mid-1970s and divided into two wings, here called Wards A (Figure 1) and B. The wards had a similar design; a central corridor with stables, examination/treatment, cleaning and storage rooms, staff and students facilities etc. on either side. The stables contain one to eight stalls with an entrance from a yard shared between Ward A and B and one from the central corridor. Floors and main walls were made of rough concrete, while intersecting walls between stalls were made of rough wood in Ward A and polyethylene in Ward B.

Ward A housed the main activities, with the main entrance, front desk, day patients and surgery unit. The central corridor served as a communication and lameness run-up area, where day patients and hospitalised horses could meet (Figure 1). Stable A was the main stable for surgical aftercare (Figure 1). The stables near the main entrance housed the day patients. Isolation units (ISO) were available in both wards: in Ward A as one unit with four separate stalls, an adjoining ante-room to each stall and a common treatment area (Figure 1), in Ward B as four separate stables with one stall and ante-room in each stable. The intensive care unit (ICU) was a stable with three stalls in Ward B and, if needed, the ISO in Ward B was also used as an ICU. Ward B also housed some specialist clinics.

Sinks, liquid soap, hand disinfectant dispensers and paper towels were available in all stables, examination/treatment rooms and the ante-room to the surgery theatres. Hand disinfectant dispensers were also available at the bandage and medication cart. Equipment such as halters, halter-chain, muzzles, twitches and mucking-out items such as pitch forks, shovels, etc. were kept in the stables.

### Infection prevention and control at the beginning of the outbreak

Work to extend and improve infection prevention and control in the clinic had started about a year before the outbreak, but no written routines had yet been established. Informal routines were practised at the time of the outbreak, as briefly described below.

Working clothes were provided by the hospital, and changed when needed, i.e. when dirty or daily. Clothing routines in the surgical unit were: change from work clothes to scrub suits and designated footwear and, during procedures, disposable gowns, hats, gloves and masks.

The hair around the surgical site of the horses was removed with a disinfected close-cutting shears and the skin was washed with chlorhexidine soap (Hibiscrub^®^) and disinfected with chlorhexidine alcohol. The operation theatres were partly cleaned with water + detergent between procedures. If needed, e.g. in the case of infectious discharge, disinfectant was used after cleaning.

The surgery recovery rooms had concrete floors and paper bedding. The rooms were cleared of bedding and hosed down with water on most working days and, if needed, scrubbed with detergent. The sling (tarpaulin) carrying the patients in and out of the operating room and mattresses used on the operation table were hosed down with water when dirty. The slings were hung to dry outside the surgical unit and the mattresses hung within the surgery unit.

Suspected contagious horses were admitted to the ISO. The common practice for staff was to put on reusable protective gowns, gloves, cap and rubber boots or shoe-covers in the ante-room before entering the ISO stalls. Foot bath or mats soaked with Virkon^® ^were also used at the staff entrance to ISO and sporadically elsewhere if symptoms of contagious disease were detected after horses had already been housed in a non-ISO stall and temporary isolation was decided.

Walls and floors in wards were cleaned by staff, either hosed down with pressure washer or scrubbed by hand between patients. After a known infectious case, the cleaning was followed by foaming with Virkon^®^.

### Environmental sampling and culture

The environmental sampling scheme was a consensus decision by the community veterinarian (CV), the hospital and the author KB. The sampling was funded by the UDS.

All environmental sampling in the hospital was carried out by author KB after routine cleaning and disinfection. Sterile cloths pre-impregnated with buffered peptone solution with 10% neutralising agent (lecithin, Tween 80, L-histidine, and sodium thiosulphate), and gloves pre-packed in stomacher bags (SodiBox^©^, Névez, France) were used [38]. At the start and between each sample, the sampler disinfected her hands before putting on new gloves.

The environmental samples were divided into two categories: 1) Surfaces or equipment in contact with horses and people, named *horse contact surfaces*; and 2) surfaces or equipment where only people (predominantly staff) had access, named *hand touch surfaces*. Equipment or surfaces of a similar kind in one room or unit were pooled into one sample. For sampling of larger surfaces, such as walls in a stall, one cloth was gently rubbed in a 1-2 m^2 ^area extending from 5-20 cm above the floor to about 1.7 m high vertically at least on three of the walls. Floors were sampled by dragging the cloth over most of the area, including floor drains. Within five hours, 25 mL of Mueller-Hinton (MH) broth with 6.5% NaCl were added to the stomacher bag containing the cloth from the environmental sampling, the contents were carefully mixed for 1 minute and the stomacher bag was incubated overnight at 37°C. On the next day, approximately 1 mL of the broth was inoculated into 9 mL Trypton soy broth (TSB) containing 4% NaCl, 1% mannitol, 16 mg/L phenol red and cefoxitin 3.5 mg/L (Sigma-Aldrich, Steinheim, Germany) and aztreonam 75 mg/L (Bergman Labora, Danderyd, Sweden) and again incubated overnight at 37°C. Approximately 10 μL of the TSB were then plated onto Brilliance MRSA agar, (Oxoid, Basingstoke, UK) with 5% bovine blood agar and incubated at 37°C for 24 to 48 h.

### Horse sampling and culture

Horses were sampled in nose or wound. Swabs containing Amies transport medium (Venturi Transystem ^®^, COPAN, Italy) were used for sampling.

Samples from horses were analysed on the day of sampling or the following day. The swab tip was clipped with disinfected scissors, placed in 10 mL Trypton soy broth (TSB) tubes containing 4% NaCl, 1% mannitol, 16 mg/L phenol red and cefoxitin 1 mg/L (Sigma-Aldrich, Steinheim, Germany) + aztreonam 50 mg/L (Bergman Labora, Danderyd, Sweden) and incubated overnight at 37°C. Approximately 10 μL of the TSB were plated onto a Brilliance MRSA agar with 5% bovine blood agar (SVA) and incubated at 37°C for 24 to 48 h.

Phenotyping of suspected *S*. *aureus *isolates was performed as described previously [37].

### Personnel sampling and culture

Voluntary MRSA testing of staff (nares, perineum) was offered in September 2008. Sampling of people with skin lesions and in contact with infected horses was recommended by the Public Health. Samples from humans were examined by an accredited Laboratory of Clinical Microbiology.

### Genotyping

All isolates from environmental samples were *spa*-typed at the Swedish National Veterinary Institute (SVA) as described by Harmsen et al. [39]. Macro-restriction analysis by PFGE was also carried out by SVA, using the enzymes *Cfr*9I, a neoschizomer of *Sma*I, and *Apa*I according to the HARMONY protocol [40]. Chromosomal DNA of *S. aureus *NCTC 8325 was used as reference size markers for normalisation of PFGE gels. Banding patterns were compared visually and the level of similarity between patterns used for defining pulsotypes was a minimum of one band difference [41].

Genotyping of isolates from the outbreak horses is described in a previous study [37].

### Statistics

Descriptive statistics and individual results are presented.

## Results

### Initial planning

Table 1 summarises the course of events in chronological order. The first intervention by the UDS was to suspend elective surgery on July 4, the same day as the second MRSA case was diagnosed. Stable A was closed for sanitation and positive horses still in hospital were moved to the ISO unit. Veterinary authorities at the regional and national levels and the Public Health authority directly started to discuss necessary measures through telephone consultations. The authorities involved were the SJV, SVA, the CV and Public Health. The CV is responsible for contagious disease in animals in the region. International contacts were established to collect information from countries with higher prevalence and experience of MRSA in horses. Representatives from the hospital and the authorities had a planning meeting at the hospital at the request of the UDS and on the initiative of the CV (Table 1, Meeting 1). The interventions decided at this meeting were: i) Phone tracing of all horses operated on during the outbreak (May 22 to July 10) in order to locate infected horses at home and culture those for MRSA [37]; ii) Interior environmental sampling for MRSA (Table 2, Figure 1); iii) Consultation of expertise in human infection prevention and control (IC); and iv) Purchase and installation of hand disinfection dispensers at multiple strategic points. Other meetings followed for feedback, discussions and reconsideration of plans. Minutes of the meetings were kept.

**Table 2:** Scheme and results of environmental sampling for MRSA on nine occasions in an equine hospital

Setting	Sampling area	Sample cat. ^4)^	2008 July 25	Nov 19	Dec 1	2009 June 8	Sep 29	Oct 15	2010 Mar 4	Mar 17	Apr 15	Total
**Ward A**												

**Stable A -** surgery patients	Walls + floor	1	0/1 **^7)^**	0/1	- - **^5)^**	0/1	- -	- -	0/1	- -	- -	0/4
	Water feeders	1	0/1	0/1	R **^6)^**	R	R	R	R	R	R	0/2
	Water buckets	1	- -	0/1	- -	0/1	- -	- -	- -	- -	- -	0/2
	Muzzles + twitches	1	0/1	0/1	- -	- -	- -	- -	- -	- -	- -	0/2
	Salt stones + cribs	1	**1/1**	R	R	R	R	R	R	R	R	**1/1**

**Room 3 ^1)^**	Light switch + door knobs	2	0/1	0/1	- -	0/1	- -	- -	- -	- -	- -	0/3

**Surgery unit**	Tarpaulins + traverse	1	**1/1**	0/1	R	R	R	R	R	R	R	**1/2**
	Recovery room 1	1	0/1	0/1	- -	0/1	0/1	- -	0/1	- -	- -	0/5
	Recovery room 2	1	- -	- -	- -	- -	- -	- -	0/1	- -	- -	0/1
	Prep. stall + tracheotube	1	0/1	0/1	- -	0/1	0/1	- -	0/1	- -	- -	0/5
	Mobile operating table	1	0/1	0/1	- -	- -	0/1	- -	0/1	- -	- -	0/4
	Surgery equipment	1	- -	- -	- -	- -	0/1	- -	- -	- -	- -	0/1
	Surgery equipment	2	0/1	0/1	- -	**1/1**	0/3	- -	0/2	- -	- -	**1/8**

**Lameness run-up**	Door knobs (n = 21)	2	**1/1**	**1/1**	0/7 **^8)^**	0/4 **^8)^**	- -	- -	0/1	- -	- -	**2/14**

**ISO ^2)^**	Ante-room	2	**2/2**	- -	- -	0/2	- -	0/2	- -	- -	0/1	**2/7**
	Stall, walls + floor	1	0/1	- -	- -	- -	- -	- -	- -	- -	- -	0/1
	Stall, floor	1	- -	- -	- -	- -	- -	0/1	- -	- -	- -	0/1
	Stall, walls	1	- -	- -	- -	- -	- -	0/1	- -	- -	**1/1**	**1/2**
	Treatment area	1	0/1	- -	- -	- -	- -	0/1	- -	- -	0/1	0/3
	Treatment area	2	- -	- -	- -	0/1	- -	- -	- -	- -	- -	0/1

**Day patient** **area**	Treatment room 1	1	- -	- -	- -	- -	- -	- -	0/1	- -	- -	0/1
	Treatment room 2	1	- -	- -	- -	- -	- -	- -	0/1	- -	- --	0/1
	Treatment room	2	- -	- -	- -	- -	- -	- -	0/1	- -	- -	0/1

**Ward B**												

**Room 4**	Treatment stall	1	**1/1**	0/1	- -	0/1	- -	- -	0/2	- -	- -	**1/5**

**Stable K -** **ICU ^3)^**	Walls + floor outside stall	1	- -	- -	- -	- -	0/1	- -	0/1	0/1	- -	0/3
	Walls + floor inside stall	1	**1/1**	0/1	- -	0/1	0/1	- -	0/1	0/1	- -	**1/6**
	Outside stall	2	0/1	0/1	- -	0/1	- -	- -	- -	- -	- -	0/3

**Equipment**	Medicine cart	2	- -	- -	- -	- -	0/1	- -	- -	- -	- -	0/1
	Bandage cart	2	- -	- -	- -	- -	0/1	- -	- -	- -	- -	0/1
	Dental equipment	1	- -	- -	- -	0/1	- -	- -	- -	- -	- -	0/1

		**Total**	**7/18**	**1/14**	**0/7**	**1/17**	**0/11**	**0/5**	**0/15**	**0/2**	**1/3**	**10/92**

Equipment or surfaces of a similar kind in one room or unit were pooled into one sample. For example, a number of door knobs/water feeders/salt stones + cribs were wiped with one cloth, making one pooled sample

**^1) ^**standing surgery; **^2) ^**isolation unit; **^3) ^**intensive care unit; **^4) ^**denotes sampling category, *horse touch surfaces *= 1 and *hand touch surfaces, where no horse had access *= 2, **^5)^**- - = not done, **^6) ^**R = replaced, **^7) ^**Number of positive/total number of samples. ^8) ^The 21 doorknobs divided into seven and four samples, respectively

### Interventions

An infection control committee consisting of the IC consultant, representatives from the UDS management and staff, the CV and SVA officials and an executive working group with the IC consultant, a nurse responsible for hygiene and a veterinarian was formed to develop and implement routines and to work with the staff. Lectures and teaching sessions on basic hygiene precautions as well as basic information about MRSA and other resistant bacteria were given to all categories of staff.

Policy documents (written routines) were developed and implemented during the seven months after the outbreak and the documents were made available to all at the hospital. The written routines were: 1. Basic personal hygiene policy. 2. Hygiene in surgery unit. 3. General surgery unit routine including cleaning, instrument washing and disinfection, and preparation of horses for surgery. 4. Cleaning routine: ISO. 5. Cleaning routine: day patient area. 6. Cleaning routine: remaining stables 7. Cleaning and disinfection: flexible endoscope.

The main issues of the written routine 1. Basic personal hygiene policy, were: i) Wrist watches, rings and other jewellery not allowed to be worn in clinical work. ii) Long hair to be tied back, nails cut short and free from coloured nail polish. iii) Hand hygiene; in the case of visible dirt, washing with soap and disinfecting hands between patients and/or different procedures; if no visible dirt only disinfection between patients or procedures. iv) Disposable gloves to be used in contact with pus, secretions, blood and other potentially contagious material. Hands disinfected before putting on and after taking off gloves. v) Working and protective clothing to be worn, as described in more detail below. Members of staff as well as teachers and students were required to read and sign their own copy of the document.

No private clothes were allowed at work. All members of staff were provided with short-sleeved working clothes, worn only at work and changed daily or earlier if dirty. Aprons for protection of the working clothes were made available in treatment rooms and stables. Fleece vests to be worn on top of short-sleeved clothes for warmth were offered to the staff. Outdoor work jackets were allowed, as long as the basic hygiene routines were followed and special holders for the jackets were installed. At the ISO unit, disposable protective overalls/gowns, clothes, caps and boots were installed in the preparation room, to be donned before entry to the isolated horse.

Disposable overalls, caps and clean shoes had to be donned before entry to the surgical unit and clothes used outside the unit were prohibited. Staff working in the surgery unit was provided with special working clothes.

Wound care hygiene was improved through implementation of contact precautions following the 'Basic personal hygiene policy' and all waste after wound care had to be put directly into plastic waste disposal bags. Sampling for culture of all patients with SSI or traumatic wound infection before any treatment started was implemented as a routine. Work to devise better routines for wound care by drainage, gentle cleansing, different dressings, less use of antimicrobials and formulating a written wound care policy is still ongoing.

Multiple dispensers with alcohol hand-rub were placed at strategic points, beside all doors and sinks, easy to find and use. Disinfectants had to be kept in their original packaging. Extra clocks were bought and mounted on walls, as well as watches to pin on working clothes to replace wrist watches.

The cleaning routines could not be much improved in the wards, as most of the surfaces were rough and manual cleaning difficult, but one stable at a time was closed for incoming patients and cleaned. Salt stones, cribs and water feeders in stalls that could not be cleaned were removed and replaced with buckets. A flusher-disinfector was installed for emptying and cleaning buckets between patients. Loose equipment such as twitches and muzzles were removed from the stables and a routine to change the twitch rope and clean and disinfect twitch and muzzle between patients was implemented. The routines for handling, cleaning and disinfection of endoscopes were devised with the aid of the endoscope manufacturer, resulting in less damage and better maintenance for bronchoscopes and arthroscopes.

The walls, floors and other surfaces of the surgery theatres were thoroughly cleaned by professional cleaners trained at Uppsala University Hospital, using high pressure washing and floor cleaning machines, before restart of surgery after the outbreak. New manual cleaning routines, including manual scrubbing of walls and floors with appropriate detergents as well as wiping when feasible, were implemented. Operating tables were covered with disposable polyethylene sheets commonly used by construction companies. Slings used to transport anesthetised horses in the surgical unit were eliminated and leg lifts were implemented. Equipment used near the patients was wiped down with alcohol + detergent between patients. Shavers were disassembled and manually cleaned/disinfected. The sterilising area was equipped with a flusher-disinfector and washable anaesthetic equipment and a drying cabinet were installed. The functioning of the autoclave and washer-disinfector was thoroughly checked by the manufacturer. The surgery recovery rooms were re-surfaced with a smooth, cleanable rubber mat on floors and walls, and paper bedding was no longer used.

### Patient movements

The number of patients treated at the UDS increased from 4268 in 2007 to 6528 in 2009. From May 1 to Aug 31 2008, 1736 horses passed through the hospital (not including radiology patients referred from outside the UDS) and between 22 May and 10 July 2008 (outbreak period), 45 horses underwent surgery. The suspension of elective surgery during the outbreak resulted in a temporary decrease in surgery procedures from an expected 30-40 per month to 10-14. Day patients and emergency surgery were kept at same level throughout the outbreak. Patient movements were better tracked and documented. Horses with infected wounds and other contagious infections were more strictly isolated. The building design did not allow for major changes in the distribution of patients within the hospital.

### Costs

The costs for cleaning, renovation, installation of new equipment and implementation of new routines etc. were an estimated 1.2 million SEK or around 120,000 Euros at the approximate exchange rate during this period, e.g. expenditure on hand disinfectants and disposable gloves doubled from 2008 to 2009.

### Environmental sampling and culture

Environmental samples were collected on nine occasions between July 2008 and April 2010 (Table 2 Figure 1). The first sampling was carried out about two weeks after the second MRSA case had been identified. The second and third samplings were performed when interventions had been implemented and embedded (Table 2). Several door knobs or water feeders or salt stones + cribs were wiped with one cloth, giving pooled samples for these types of objects (Table 2).

Ten (11%) of 92 samples were MRSA-positive. Seven of these came from the first sampling occasion (Table 2). MRSA was found equally in both of the predetermined sample categories: *horse touch surfaces *(n = 5) and *hand touch surfaces *(n = 5). The sampling scheme from July 2008 was approximately the same on three more occasions (Table 2), but in November 2008 water feeders and most of the cribs had been replaced by buckets, which were sampled instead. A pooled sample from 21 door knobs in the lameness run-up corridor tested positive twice. In December 2008 all door knobs were retested a third time, in which they were divided into seven samples, with negative results. In June 2009 further sampling was performed. One computer keyboard that had not been cultured before was included in the pooled sample of *hand touch surgery equipment*, and tested positive for MRSA (Table 2). In September 2009, 11 pooled samples were taken, including the *hand touch surgery equipment *that tested positive in June, and all tested negative (Table 2).

Environmental sampling with fewer samples was carried out on 15 October 2009 and 17 March and 15 April 2010 in the surgery unit and ISO when two sporadic MRSA cases had been detected in September 2009 and March 2010. With these samples, there was one positive finding from an ISO stall where an MRSA-positive horse had been housed (Table 2).

### Horse sampling and culture

Fourteen asymptomatic contacts in the home stables of Cases 1, 2 and 3 were cultured from the nares, and one tested positive for MRSA *spa*-type t011. Forty-five horses underwent surgery in the period May 22-July 10 2008. The owners of 37 of these were reached by telephone, and four reported horses with symptoms of infection (secretions, swelling, etc.) at the surgical site. Three of these were cultured, with one MRSA-positive result (Case 5).

### Personnel sampling and culture

One-third (12/30) of the hospital staff as well as four people with skin lesions linked to positive infected horses at home were tested for MRSA, and all tested negative (Mia Runnerus, director of UDS, pers. comm.).

### Genotyping

**Figure 2:**
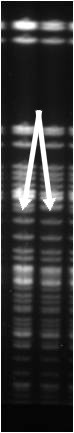
**PFGE pattern with *ApaI *restriction enzyme showing different pulsotypes of environmental MRSA isolates *spa*-type t011**. One band of difference (arrow), Right lane - pulsotype A. Left lane - pulsotype A1.

All environmental isolates were of *spa*-type t011, i.e. the same as the isolates from the outbreak horses [37]. The PFGE pattern was identical with both *Cfr*9I and *Apa*I restriction for nine of 10 isolates, named pulsotype A. The tenth isolate, the last one found in April 2010, lacked one PFGE band in the *Apa*I gel, pulsotype A1 (Figure 2).

## Discussion

The University Animal Hospital offers highly qualified surgery and medicine and some patients are immunocompromised. Consequently, the usage of antimicrobials might be relatively high. Since antimicrobial usage has been described as a risk for equine MRSA colonisation [11,42] effective infection prevention and control are essential in such settings. Success in short-term eradication by sectioning, improved infection control and repeated MRSA testing without using antimicrobials in two Canadian farms with MRSA-colonised horses has been reported [43]. The main interventions in the present study were aimed at improved infection prevention and control, supported by environmental sampling.

The hospital was expanding at the time of the outbreak, with an increase of about 1000 patients per year. Increased patient flow with more horses housed in the hospital may have led to more indirect and perhaps also direct contacts between horses and thus a higher risk of transmission of infections. Increased crowding was also considered a risk of undesirable doping of horses by pharmaceutical residues in the environment. Therefore some precautions regarding handling of horses in the hospital had been applied, although not documented, before the MRSA outbreak.

There was a lag of over a month between surgery and diagnosis of the two first MRSA cases and thereby a delay in identification of the MRSA outbreak (Table 1). The source of the outbreak was never found, since the first three horses infected had indirect cross-contacts within the hospital [37]. The immediate suspension of elective surgery when an outbreak was suspected resulted in a temporary reduction in surgical patients. This gave space and time for necessary interventions in the surgery unit and probably prevented further spread of the MRSA strain. Emergency surgery continued, for animal welfare reasons. After elective surgery was suspended, only one horse, an emergency surgery patient, was infected by MRSA. The closure of Stable A, where Cases 1-5 were housed, was logical, as it could be expected to be highly contaminated with MRSA. This was confirmed by the first environmental sampling (Table 2).

Representatives from different disciplines, the CV, a public health nurse and the SVA visited the UDS when an outbreak was suspected and started a process of change. Meeting face-to-face, walking around the hospital setting and discussing emerging issues from different angles in a mutual conversation opened the way for systematic work. Public health aspects had to be considered, as MRSA is a zoonosis [12,15-17,21,22] which is notifiable for humans, and prevention of MRSA transmission between animals will reduce the risk for humans too.

The executive working group transferred some general infection prevention and control measures from human medicine, such as basic hygiene. Further interventions undertaken to curb the outbreak were based on this general knowledge and on observed practices and shortcomings, as well as on environmental sampling.

In order to improve the guidelines for handling and interventions in outbreak situations, knowledge of risk factors for acquisition and spread of MRSA in horses is needed. Horses colonised with MRSA on admission were more likely to suffer from clinical infection than non-colonised horses in one Canadian study [11]. In a study of MRSA outbreaks in a Dutch equine hospital, 9.3% of horses tested positive on admission, and the same *spa*-types were detected in infected horses [12]. Such studies could speak in favour of screening on admission, but cannot effortlessly be transferred to low prevalence countries, as Sweden. Admission to neonatal intensive care was another identified risk factor for MRSA colonisation [42]. About a year after the outbreak, a foal was admitted for neonatal care and MRSA was later detected in a pressure wound. The MRSA status of the foal and its mother on admission was not known. On readmission for infection in September 2009, the foal was isolated before culture and the strain did not cause further infections in other horses. Sampling of horses on admission to hospital had not been considered relevant prior to the outbreak described, as only one MRSA horse had been detected earlier in the country, a carrier in 2007 [18]. At present there is still no general screening for MRSA in horses on admission to the UDS because of the low prevalence, although horses with discharging wounds are sampled.

Culture is time-consuming and therefore fast and reliable detection methods for screening on admission are needed. A real-time PCR validated for diagnostic purposes in humans has been evaluated, but proved unsuitable for use in horses [44]. Screening of incoming risk patients might be recommended, e.g. horses with infected wounds, previously known MRSA infection/carriage, coming from high prevalence areas or hospitals with a known outbreak etc., but only if all these patients could be isolated prior to the sampling results. Studies in humans have shown that isolation of a patient only when a positive result is obtained does not reduce transmission to other patients if basic precautions are not consistently taken [45].

Screening cultures are not wholly reliable and may give a false sense of security. There might even be a risk of staff handling screened horses testing negative with less care if horses are divided into MRSA-positive and negative groups, and thus the risk of contagious infection could increase for the negative group. Basic precautions and good cleaning routines must be implemented throughout the year and for every patient, irrespective of culture results. Isolation in single stalls should be implemented for risk patients such as those with discharging wounds.

Screening of asymptomatic contacts can be useful for epidemiological investigations and for interventions in an outbreak. Based on the severity of the disease and the risk to animal and human populations, SJV can initiate and pay for mandatory sampling. Contact tracing of discharged horses that had been operated on during the outbreak period led to the identification of one more infected horse, Case 5 [37]. Nasal screening of the large numbers of asymptomatic horses that visited the hospital during the outbreak period would have required the owners' consent, as MRSA is not subject to mandatory tracing in animals and no resources were available for this. Therefore, there were gaps in the information and we do not know how the outbreak was maintained during the weeks between the first and second cluster of cases. Information about personnel is also lacking, as about two-thirds did not attend the sampling.

Environmental sampling was introduced in order to identify routes of indirect contact transmission. No standard or recommended method for environmental sampling of MRSA was available at the time, and the sampling and culture method was a combination of methods described in the literature and personal experience. The surfaces in equine hospitals are often rough and large areas have to be sampled and ordinary swabs or contact plates cover only limited areas. Here wiping of similar surfaces and pooling of samples was performed in order to cover as many items and as large an area as possible at a reasonable cost. The pre-impregnated cloths used have been tested in similar studies of environmental enterococci [38]. The pre-impregnation neutralise possible residues of cleaning and disinfection agents. The sensitivity and specificity of the method is not known, as there is no 'gold standard'. Our culture method, with high-salt pre-enrichment broth and a second specific broth, was similar to the more sensitive method identified by v. Duijkeren et al. when comparing two methods [12]. The majority of the positive samples in the UDS were found in connection with the outbreak and the number of positive samples decreased as expected after interventions were implemented (Table 2). The second sampling (Table 2) was carried out after major interventions had been initiated. Dividing the twice positive 21 doorknobs pooled sample into seven pools, when retesting 1 December 2008, were made to check if many doorknobs might be positive, but this time tested negative. Some time was then allowed to elapse for the interventions to bed down before the third sampling in June 2009, as the main purpose of repeated sampling was to monitor improvements.

The MRSA isolates found in the environment had the same PFGE pattern as the isolates from the outbreak horses described earlier [37]. The isolate from last environmental sampling, in April 2010, showed a slight genetic change in PFGE, pulsotype A1, compared with the nine previous isolates (Figure 2). This isolate was found in an ISO stall after a horse with the same pulsotype (A1) had been discharged. The isolate was still considered to have the same origin as the other nine. It is not uncommon for bacterial strains to change genetically over time and a one to two band difference in PFGE is considered to be closely related [41].

In order to distinguish between human contamination and horse/human contamination, the sampling surfaces were divided into two categories, *horse touch surfaces *and *hand touch surfaces *(Table 2). The results of the environmental sampling were used for educational purposes, to increase awareness of basic hygiene, and for the planning of interventions. Findings of the MRSA strain on *hand touch surfaces *indicated spread by humans (Table 2 Figure 1). The finding of MRSA on door knobs (Table 2 Figure 1) led to the installation of dispensers with alcohol hand-rub beside all doors, with the idea 'easy to find and do right'. Other ways of improving contact precautions (basic hygiene) were better clothing routines, the increased use of plastic aprons and gloves, and the replacement of disinfectant shoe baths/mats with disposable boots for isolation stalls.

The finding of MRSA in a sample from salt stones and cribs supported the decision to remove those and the water feeders and replace them with buckets. Buckets were easy to clean in the installed flusher-disinfector and they tested negative for MRSA on two occasions (Table 2). An additional benefit was better water control for the patients. As MRSA was found in the pooled sample of the sling (tarpaulins) and traverse in the surgery unit, these were removed. Using disposable plastic sheets on the mobile operation tables was the unconventional solution introduced to keep the costs for disposable equipment at reasonable levels.

*Staphylococcus aureus*, including MRSA, can persist on dry inanimate surfaces for months [46], and cleaned surfaces are rapidly recontaminated when infective patients are present. Therefore, the findings of MRSA where infected horses had been stalled were not surprising. Written disinfection and cleaning routines available to all were developed and replaced long-standing procedures for environmental cleaning and disinfection. Qualitative environmental sampling is not considered sensitive enough to validate cleaning routines and should not be used for that purpose [36]. Despite this, in the present outbreak negative environmental cultures tended to give staff a false sense of security, as they tended to interpret these as successful eradication of MRSA.

Some items were more difficult to improve, e.g. floor coverings, which need to be rough for the safety of the horses but then become difficult to clean. The walls and floors in the surgery recovery rooms were worn out and considered a high risk for the spread of MRSA and were replaced with non-porous and easy to clean material. The remaining floors were not changed, since the outbreak coincided with construction plans for a new hospital building. Improvement of infection prevention and control in the daily clinical routines was gradually achieved but the conditions in the building were a limiting factor for cleaning.

The doubling in expenditure on gloves and disinfectants is a reasonable indication of improved standards of hygiene. On the other hand, MRSA was still found on surfaces that were not directly available to horses long after the last outbreak case of MRSA. Thus, compliance with basic hygiene and/or cleaning and disinfection routines has improved but is still not complete and requires continual follow-up to ensure maximum effect. Methods to measure compliance with hygiene routines need to be introduced in veterinary practice. Observational studies are a possibility to record improvements in hygiene routines. Quantifying the efficacy of the interventions would also be advantageous, but was not the aim of this study.

The interventions led to a reduction in MRSA-positive samples, and no new cases occurred for over a year, despite the extended presence of a colonised horse (Case 6). The case detected 14 months after the outbreak, a foal admitted for neonatal care in June 2009, had the same MRSA strain as the six outbreak horses in 2008 [37]. The strain was also found on *hand touch surfaces *in the surgery unit in June 2009. Whether this was the cause or the effect of the presence of an infected/colonised horse could not be determined, as we do not know whether the foal was carrying the strain on admission or became infected within the hospital. No other MRSA infections were detected in connection with this case. The case that occurred in March 2010 was also a single case without any other detected infections in the hospital. It is unlikely that pathogenic MRSA would remain undetected, as all infected wounds were cultured. The MRSA ST398, *spa*-type t011, is common outside Sweden [12,13,15,16]. A closely related strain caused an infection linked to another equine hospital in the area in 2009 [37]. This indicates that the strain is present in the region outside the hospital studied here. We therefore consider Case 6 to be the last case in the outbreak and the other two later cases as sporadic.

The costs of an outbreak will differ between clinical settings and can be divided into different categories in order to have clear view of expenses: i) Costs directly linked to an outbreak, exemplified by suspension of elective surgery, extra working hours, isolation of horses, damage to reputation resulting in a future decrease in patients, bacteriological sampling, hire of a cleaning team and IC expert; ii) running costs to achieve general high quality, exemplified by increased expenditure on disposable gloves, other disposable items, soap, disinfectants, clothes, continuous education and revision of routines; and iii) depreciable investments, exemplified by renovation of recovery rooms, purchase of washer and flusher-disinfectors, changing from water feeders to buckets.

Distinction of costs into these categories was not done at the UDS. The calculation of total cost resulted in a rather high figure, but depreciable investments were included and it is debatable whether such investments should be fully included as costs due to an outbreak. Direct costs and running costs might also have been under- or over-estimated, as they are difficult to calculate exactly. If detailed distinctions had been made during the process, it would have been easier to analyse the cost elements justified for future infection control.

This outbreak and earlier incidents of MRSA in pets, mainly dogs, have resulted in growing interest in infection prevention and control policies by Swedish animal hospitals and clinics. MRSA has been notifiable since 1 January 2008 in animals. Guidelines for owners of MRSA-positive horses were published on the SVA website in July 2008. A Swedish Government Commission draft strategy from March 2011 against antimicrobial resistance and nosocomial infections drawn up by the National Board of Health and Welfare (Socialstyrelsen) includes veterinary medicine [47]. Infection prevention and control has been introduced as a topic in the Veterinary Nursing programme at the Swedish University of Agricultural Sciences since 2009. This is an important step towards a more professional view of veterinary medicine infection prevention and control for the future.

The outbreak had consequences outside the hospital and authority sphere, as in 2009 the Swedish Equestrian Federation and the Swedish Horse Racing Totalisator Board introduced a competition ban for horses with MRSA infection and 20 days after healing of the infection:http://www3.ridsport.se/Tavling/Vill-du-borja-tavla/Smittinfo/ and https://www.travsport.se/polopoly_fs/1.574!/menu/standard/file/smittskyddsreglement2010.pdf

## Conclusions

Joint efforts by hospital management, microbiology and infection control, with support from local and national authorities, curbed the MRSA outbreak studied here. The occurrence of single horses infected by the same strain and subsequent findings of the MRSA strain in the hospital and the surrounding region show the need for continuous awareness and improvement of infection prevention and control measures.

Long-term improvement of infection prevention and control in the hospital can be achieved through written policies for basic hygiene and for isolation of infected cases, as well as for improved cleaning and disinfection. Environmental samples can be useful for identification of routes of transmission, education of staff and identification of surfaces and equipment that cannot be cleaned or disinfected and should be replaced, although this is not always feasible in equine hospitals.

Knowledge of infection prevention and control, nationally and in the veterinary profession, is necessary for the prevention of future MRSA outbreaks in equine hospitals.

## Competing interests

The authors declare that they have no competing interests.

## Authors' contributions

KB initiated and wrote the study together with UR and GN. KB designed the protocol for sampling in the environment. UR was the infection control expert who led the work at the UDS. CJ was the contact at the hospital, providing the information and records and adding editorial comments to the manuscript. SW designed Figure 1 and provided information from the process at the time of the outbreak. UGA contributed the authority view to the manuscript. All authors have discussed, read and agreed the final manuscript.
